# Insufficient Logging Intervals Impede Upper Soil Recovery in Temperate Beech Forests: Insights From Two Case‐Studies in Poland

**DOI:** 10.1002/ece3.72302

**Published:** 2025-10-12

**Authors:** Francesco Latterini, Paweł Horodecki, Marcin K. Dyderski, Jacek Kamczyc, Radosław Witkowski, Rachele Venanzi, Andrzej M. Jagodziński

**Affiliations:** ^1^ Institute of Dendrology Polish Academy of Sciences Kórnik Poland; ^2^ Department of Game Management and Forest Protection, Faculty of Forestry and Wood Technology Poznan University of Life Sciences Poznan Poland; ^3^ Department of Forest Entomology and Pathology, Faculty of Forestry and Wood Technology Poznań University of Life Sciences Poznań Poland; ^4^ Department of Agriculture and Forest Sciences (DAFNE) University of Tuscia Viterbo Italy

**Keywords:** forest operations, litter decomposition rate, QBS‐ar index, recovery time, soil compaction, timber harvesting

## Abstract

Little is known about how forest operations affect the biodiversity of soil microarthropods and the litter decomposition rate in temperate beech forests. This study aims to ascertain this information. Two study areas were selected, each consisting of a chronosequence of three cutting blocks: one that had not been harvested in the previous 20 years, one harvested in 2017 and one harvested in 2021. In 2022, we examined skid trails in the harvested parcels, categorised as disturbed soil and soil that has not been impacted by any machine passage, categorised as undisturbed soil. There were five experimental treatments in total within each study area, including the control. For every treatment, we evaluated upper soil compaction, organic matter content and soil microarthropod biodiversity, which was measured using the QBS‐ar index. To compare the variations in litter decomposition rates among treatments, we also set up a litter decomposition experiment based on the teabag method. Aside from the litter decomposition rate, which remained unaffected in all experimental treatments, we identified significant disturbances in the soil impacted by the machine's passage. Our results suggest that the recovery process for all variables studied was still incomplete after 5 years. Skid trail sites established 5 years ago continued to display values that differed from those in undisturbed and control areas. We recommend increasing the time interval between two consecutive logging operations in the same cutting block or implementing best management practices that can reduce the initial disturbance in the skid trails.

## Graphical abstract

This study examined the effects of forest operations on soil microarthropod biodiversity and litter decomposition in temperate beech forests, comparing disturbed and undisturbed soils across different harvesting timelines. While litter decomposition remained unaffected, significant soil disturbances were found in areas impacted by machine passage, with recovery still incomplete even after 5 years. The findings suggest extending the time between logging operations or adopting best management practices to minimise initial disturbances in skid trails.

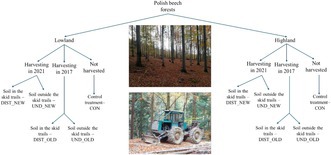

## 1 Introduction

Safeguarding soil health and ensuring soil quality have been identified as pivotal issues in the European Union agenda (European Commission 2021; Vanino et al. 2023; Warnke et al. 2023). Logging operations are known to represent a potential threat to the forest soil, mostly when using ground‐based logging systems (Labelle and Jaeger 2011, 2012; Jiang et al. 2023). The first direct consequence of a machine trafficking the forest is generally a temporary alteration of the soil structure, generally observable as an increase in the soil bulk density (BD; Guimarães Júnnyor et al. 2022; Nazari et al. 2023; He et al. 2024) and in the formation of ruts (Mohieddinne et al. 2022) along the skid trail network. Such disturbed soil can cover from 10% to 30% of the overall surface of the logged cutting block (Klein‐Raufhake et al. 2024; Latterini, Dyderski, et al. 2024). However, soil compaction and rutting only represent the first direct consequence of machinery‐induced soil disturbance to the forest soil, considering that it can also imply an alteration to several biological features of the soil (Liu et al. 2023; Ni and Su 2024; Klein‐Raufhake et al. 2024). In the soil affected by the passage of the machine, gas exchange is generally altered, with reduced oxygen availability (Epron et al. 2016), increased carbon dioxide concentration (Ampoorter et al. 2010) and methane emissions (Vantellingen et al. 2022). Several studies carried out in the framework of the Mediterranean forestry context revealed a significant reduction of soil microarthropod biodiversity in the skid trails in different forest stand types (Latterini, Venanzi, et al. 2023; Latterini, Horodecki, et al. 2024).

Although machinery‐induced soil disturbance has received considerable attention in recent years, significant research gaps remain, particularly in European broadleaf forests and especially in beech stands ( *Fagus sylvatica* L.), a key species in European silviculture (Antonucci et al. 2021; de Tomás Marín et al. 2023; Muffler et al. 2024). Extensive research concerning logging disturbance to beech forests has been carried out in Iran, focusing on the alteration of soil physical properties after ground‐based skidding when applying the single or group selection methods (Naghdi and Solgi 2014; Naghdi et al. 2015, 2016). It is important to consider that studies concerning beech forests in Central Europe, which are generally managed with the shelterwood method, are scarce and focused mostly on the soil physical properties, that is, soil compaction (Rohand et al. 2003). Most importantly, there is no study which evaluates the alteration that logging disturbance can induce on soil microarthropod biodiversity in temperate European beech forests. Furthermore, the few studies that considered this topic in other parts of the world generally did not focus on the recovery time after harvesting and just evaluated the short‐term disturbance (Latterini, Venanzi, et al. 2023; Latterini, Dyderski, et al. 2024). Studies from different parts of the world highlighted that the process of recovery of the soil in the skid trails is strongly connected to the magnitude of initial disturbance (DeArmond et al. 2024). Decades can be needed for heavily trafficked trails and landing sites (DeArmond et al. 2022), while the process can be much faster (about 5–10 years) in secondary and tertiary trails which experienced a lower level of machinery traffic (Venanzi et al. 2019; Latterini, Horodecki, et al. 2024).

Taking the above into account, there is a lack of data assessing whether current management practices in temperate beech forests, and the intervals between consecutive logging operations within the same cutting block, align with the time required for the soil to recover its physicochemical and biological properties (DeArmond et al. 2021). What is more, there is a complete lack of information concerning the effects of logging operations on litter decomposition rate in such stands, as highlighted by our recent meta‐analysis (Latterini, Dyderski, et al. 2023). Enez et al. (2015) found that only heavily scalped soil after winching operation by a forestry‐fitted farm tractor caused a significant decrease in litter decomposition rate. Furthermore, Kranabetter and Chapman (1999) and Yoshida et al. (2019) revealed that soil that was compacted by crawler tractors or excavators did not exhibit significant changes in the litter decomposition rate. Consequently, no information is available at this time regarding the ways and degree to which logging operations in beech forests could modify the process of litter decomposition in the skid trails.

Recognising the significant gaps in our understanding, this study sought to pioneer research into the effects of logging on temperate European beech forests. Our objectives encompassed a comprehensive evaluation of disturbances to soil physico‐chemical properties, microfauna biodiversity, and litter decomposition dynamics. Furthermore, we evaluated the recovery process after harvesting, trying to understand if the current management schedules are in line with the needs of the forest soil. We hypothesised that: (a) soil physico‐chemical features and soil microfauna biodiversity are significantly altered in the soil affected by machine passage; (b) as a consequence of the slight canopy alteration after thinning, the soil physico‐chemical and biological features are not altered in the soil not affected by machine passage; (c) a 5‐year period is not enough to restore the soil physico‐chemical and biological features in the skid trails; (d) decreased soil microfauna biodiversity is related to decreased values of soil organic matter and increased soil compaction; (e) litter decomposition rate is slowed down in the skid trails and a 5‐year period is not enough to restore the values to the pre‐harvesting levels.

## 2 Materials and Methods

### 2.1 Study Areas and Rationale of the Experiment

We identified two study areas in Poland, one located in a lowland area in Northern Poland (Area 1—Kolbudy Forest District) and one in a mountainous area in Southern Poland (Area 2—Sucha Forest District). The studied cutting blocks in Area 1 are characterised by Arenosols with a sandy‐loamy texture; the average annual temperature is 7°C, with an average annual precipitation of 685 mm. The cutting blocks in Area 2 are characterised by silty‐loamy leptosols; the average annual temperature is 5.5°C, with an average annual precipitation of 1000 mm. Study area 1 is representative of beech forests in the Polish lowland, characterised by coarse texture soils and gentle slopes (Jagodziński et al. 2020; Poggio et al. 2021). Study area 2 is instead a typical example of beech forests in the Polish highlands, with soils with a finer texture and harsher topography (Jagodziński et al. 2020; Poggio et al. 2021), which, however, does not generally hamper the possibility of carrying out mechanised forest operations (Latterini, Jagodziński, et al. 2023).

Both study areas consist of a chronosequence made up of three beech cutting blocks, namely: one that experienced logging in 2021 (NEW), one logged in 2017 (OLD) and one unharvested in the last 20 years (CON). The study areas were selected because they are highly representative of temperate European beech forests which can be located in lowlands and highlands (Packham et al. 2012; Durrant et al. 2016) and are actively managed by the shelterwood method. Also, the selected cutting blocks are representative of the target forest typology we wanted to investigate. Specifically, all the cutting blocks examined consist of even‐aged beech forests, with beech accounting for nearly the entire stand basal area. In the blocks harvested in 2017 and 2021, the same harvesting method was employed, the Tree‐Length System, which involves felling and delimbing trees at the stump using chainsaws, followed by extraction of full‐length stems using a cable skidder. In both study areas, timber extraction was carried out with the same model of skidder: the LKT 81 ITL (93 kW engine) manufactured in Bratislava, Slovakia. This machine has an operational weight of 8.9 tons and is equipped with two winches, each with a pulling force of 80 kN. Logging operations in all cutting blocks involved thinning from below, removing approximately 35% of the standing biomass.

**FIGURE 1:**
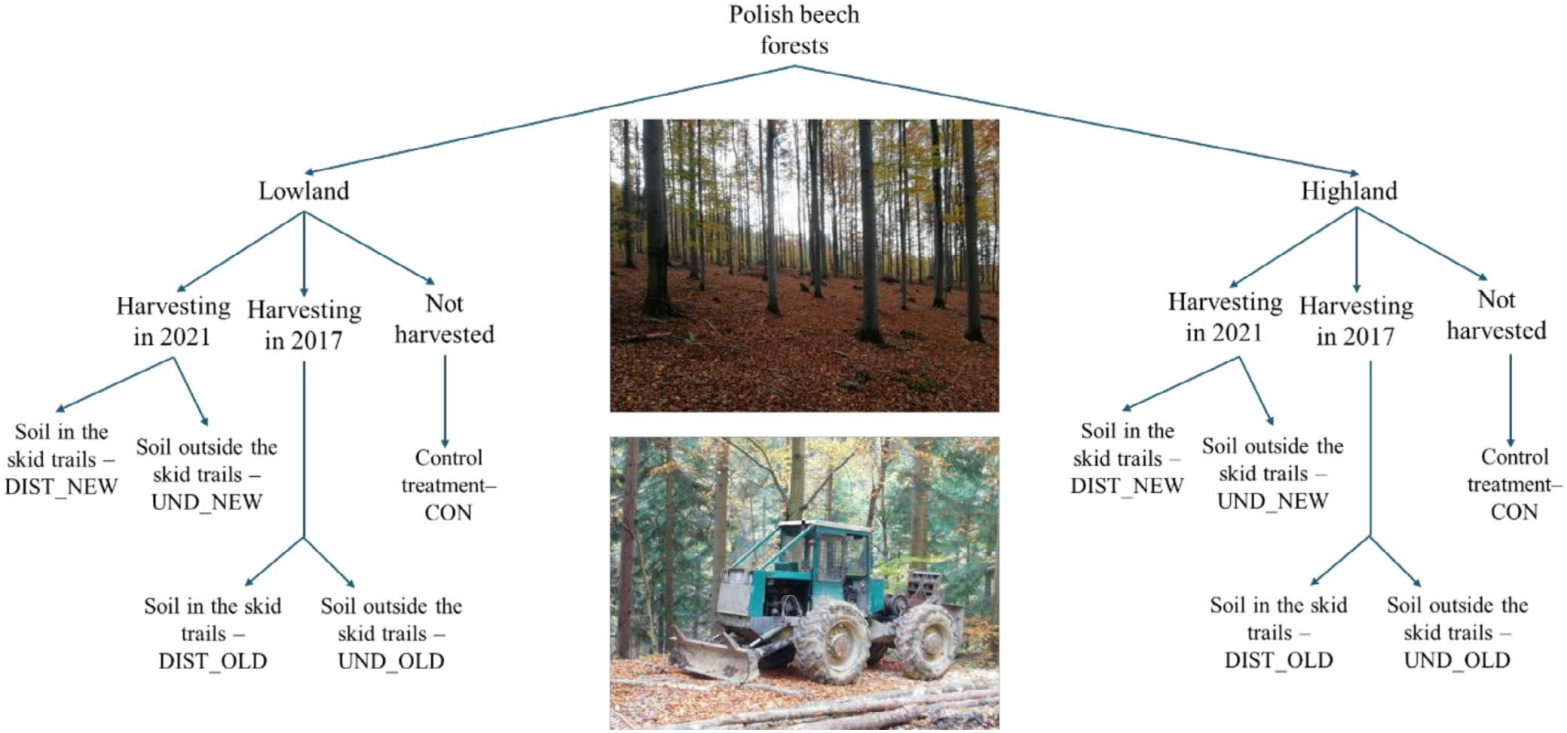
Schematic representation of the experimental design. In the centre of the figure, in the upper part, a picture of a typical beech forest in Poland; in the lower part, a cable skidder usually used for forest operations in beech forests.

In both NEW and OLD logged cutting blocks, the soil was separated into undisturbed (UND—not affected by the skidder passage) and disturbed (DIST—affected by the passage of the skidder). A schematic representation of the experimental design is given in Figure 1.

Following the methodology presented in (Latterini, Venanzi, et al. 2023), comparing the values in the disturbed soil with those in the undisturbed and control soil indicates the disturbance caused by the passage of the machinery. Comparing the values in the undisturbed soil with those in the control area provides insights into the effects of the silvicultural treatment on the soil features. Applying the same experimental design to cutting blocks logged in different years allows us to evaluate the recovery process after logging. We chose the 4‐year interval because it represents the usual time lap that occurs between two consecutive logging operations in the same cutting block in the study area contexts. Our experimental design was therefore made up of five experimental treatments: DIST_NEW, DIST_OLD, UND_NEW, UND_OLD, and CON. Considering that primary trails were practically absent in the investigated cutting blocks, we analysed only soil disturbance on secondary and tertiary skid trails (one to ten machine passes) established during the logging operations (DeArmond et al. 2021). After the end of the operations, the skid trails established during logging are closed to traffic. It is important to note that our focus was on secondary and tertiary trails, as primary trails in the study areas serve as permanent access roads to various parts of the forest districts. These primary trails are subject to ongoing traffic even after logging operations have ended, making it impractical to assess soil compaction levels on them.

### 2.2 Percentage of Surface Impacted by the Passage of the Machinery

**FIGURE A1:**
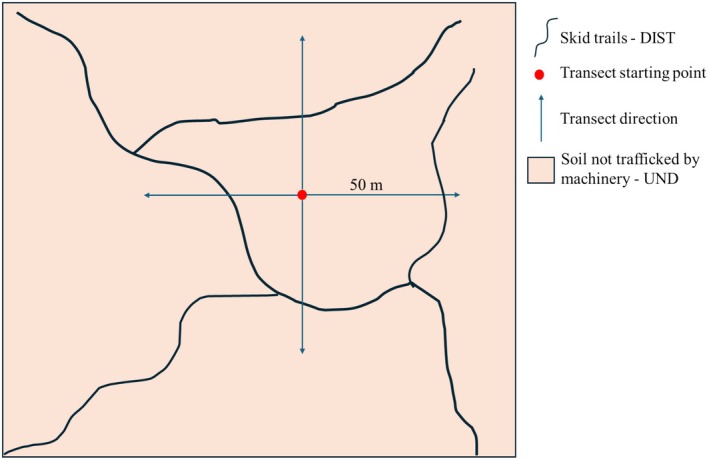
Schematic representation of the transect sampling scheme for assessing the percentage of soil area affected by the passage of the machinery.

Before collecting the data about the target soil features, we assessed the percentage of disturbed soil (DIST) relative to the total area of the cutting block. We established four linear transects (1 × 50 m) in each logged cutting block using a compass and a tape measure (Figure A1 in the attachment). Along the transect, we identified the disturbed soil by looking at crushed litter, ruts or soil mixing. Then, the disturbed surface was indicated as a percentage of the overall area of each transect. In the logged cutting blocks, the percentage of soil affected by the skidder passes ranged between 22% and 26%.

### 2.3 Soil Physico‐Chemical and Biological Features

Field surveys were conducted in October 2022 in both study areas, thus 1 year after harvesting for 2021 cutting blocks and 5 years after harvesting for the 2017 cutting blocks. Each variable was measured in specific sampling locations, allowing us to develop models linking the various soil features.

To assess the upper soil BD (5‐cm depth), we used a specialised corer (Eijkelkamp, Zevenaar, Netherlands) and we collected six soil samples for each experimental treatment in Area 1 and nine samples per experimental treatment in Area 2, resulting in a total of 75 samples. The number of samples differed between the two areas because the cutting blocks in Area 2 were larger than those in Area 1. These samples were sealed in plastic bags and sent to the lab. After oven drying at 105°C to achieve constant (dry) mass, the soil samples were weighed. Soil BD was then calculated by dividing the dry mass by the volume of the corer cylinder (100 cm^3^). Additionally, a handheld penetrometer and vane tester (Eijkelkamp, Zevenaar, Netherlands) were employed to assess shear resistance (t m^−2^) and penetration resistance (MPa) in the top 2 cm of soil. The obtained values were related to soil water‐holding capacity, following the approach described by Saxton et al. (1986). The same number of measurements as for BD were taken for both shear resistance and penetration resistance.

For the assessment of soil organic matter in the upper soil layers, we collected six soil samples per experimental treatment in Area 1 and nine samples in Area 2, resulting in a total of 75 samples. The same BD sampling tool was employed for this purpose. Subsequently, the organic matter content was calculated by subjecting the soil to a 4‐h combustion process at 400°C in a muffle incinerator, following Rosell et al. (2001).

The disturbance to the microfauna of forest soil was assessed using the QBS‐ar index, a qualitative index that evaluates the biodiversity of the microarthropod community within the soil (Parisi et al. 2005). The underlying concept is that a higher concentration of microarthropod groups specifically adapted to the soil environment indicates higher soil quality. These soil microarthropods exhibit diverse morphologies shaped by their environment, allowing them to be categorised into various biological types. Each type is assigned an ecomorphological index (EMI) score ranging from 1 to 20, reflecting the degree of adaptation. The QBS‐ar index is then calculated by summing the EMIs for each group in a given soil sample. For this study, six soil samples per experimental treatment in Area 1 and nine soil samples in Area 2 (75 in total), each measuring 10 × 10 × 10 cm, were collected using a dedicated soil corer. After transportation to the laboratory, microarthropods were extracted from the soil samples using Berlese‐Tüllgren funnels. These specimens were subsequently preserved in a solution (75% ethyl alcohol and 25% glycerol by volume) and taxonomically classified at different levels (order for Insecta, Collembola, Chelicerata and Crustacea, and class for Myriapoda) using a stereo microscope. The specimens found in each soil sample are reported in Table A1 in the attachment.

### 2.4 Litter Decomposition Rate

In our study, we aimed to assess litter decomposition rates by employing a standardised method known as the ‘teabag method’ (Keuskamp et al. 2013). We used two types of tea—green tea and rooibos tea—as reference materials. These teas decompose at different rates, with green tea breaking down rapidly and rooibos tea being more recalcitrant to decomposition (Keuskamp et al. 2013). A further advantage of the teabag method is the possibility to shorten the evaluation time in comparison to typical litter decomposition experiments based on litterbags.

We created labelled mesh bags measuring 15 cm × 15 cm from 1 mm mesh fibreglass netting. Each mesh bag contained one oven‐dried green teabag or one oven‐dried rooibos teabag. The mass of the teabags was measured by a precision scale with a resolution of 0.001 g before placing them in the mesh bags. The mesh bags were subsequently installed in the investigated cutting blocks in June 2023 and collected at 5, 13 and 22 weeks after the installation in both the study areas. The installation process was performed as follows: in both study areas for every experimental treatment (DIST_NEW, DIST_OLD, UND_NEW, UND_OLD, CON), we selected five locations which were labelled as replication ID. The mesh bags were tied to beech trees using nylon cords in the following locations: disturbed treatments (skid trails)—tied to trees along the skid trails; undisturbed treatments—tied to trees in areas unaffected by machinery passage; control area—tied to randomly selected trees in the control area. In total, we installed 300 mesh bags (300 teabags), corresponding to the combination of two study areas × five experimental treatments × three collection times × two tea types (green tea and rooibos tea) × five replications. During the collection process, each mesh bag was promptly placed into an envelope in the field to prevent any material loss. In the lab, we meticulously removed any traces of invading material, such as sand, insects or understory plants, by carefully sorting the teabags using tweezers under a dissecting microscope. Subsequently, the teabags were oven‐dried for a minimum period of 72 h. Finally, we weighed each teabag using a precision scale with a resolution of 0.001 g. This measurement allowed us to calculate the loss of mass in the tea.

### 2.5 Statistical Analysis

We used linear mixed‐effects models (LMMs) to assess the effects of the experimental treatment on the investigated soil features. The experimental treatment represented the fixed effect in the model, while the study area was included as the random intercept. Including a random intercept allowed us to account for the nested structure of the data within each study area, effectively addressing the spatial dependence among individual samples. We used the packages lme4 (Bates et al. 2015) and lmerTest (Kuznetsova et al. 2017) of the R software (R Development Core Team 2023). We presented the obtained results by analysis of variance (ANOVA) and marginal means, that is, mean predicted values for the global population, excluding random effects (Searle et al. 1980). We further applied Tukey's posteriori test to group the mean values of the experimental treatments. For marginal means calculation and posteriori tests, we applied the algorithms implemented within the emmeans package (Lenth et al. 2019). We further used LMMs to assess the relationship between the QBS‐ar index and the soil physico‐chemical variables. In this case, the QBS‐ar index was our dependent variable, soil physico‐chemical features were the fixed effects, and the study area was the random intercept. For the developed models, we calculated both the marginal and conditional coefficients of determination ($R_{m}^{2}$ and $R_{c}^{2}$), which explain the proportion of variability explained by fixed effects only and by both random and fixed effects, respectively (Nakagawa and Schielzeth 2013). To calculate the coefficients of determination, we used the MuMin package (Bartoń 2023). To visualise only the fixed effects of the models, we used marginal responses implemented in the ggeffects package (Lüdecke 2018).

We further used generalised linear mixed‐effects models (GLMMs), implemented in the glmmTMB package (Brooks et al. 2017), to investigate the effects of the experimental treatments on decomposition in tea bags. Considering that green tea and rooibos tea have different chemical compositions, resulting in different decomposition rates, we developed a separate model for the two tea types. In both GLMMs, we indicated the fraction of mass lost during decomposition as a dependent variable. The experimental treatment, the collection time and their interaction were the independent variables. Replication ID nested into the study area was included as the random intercept of the model. Considering the fractional character of mass loss, we assumed a Beta distribution of the response variable. We used the ggeffects package (Lüdecke 2018) and marginal means for treatments estimated using the emmeans package (Lenth et al. 2019) to visualise the response curves for each experimental treatment and collection time.

## 3 Results

### 3.1 Effects of the Experimental Treatments on Soil Physico‐Chemical and Biological Properties—Hypotheses a, b and c

The obtained results showed statistically significant effects of the experimental treatments on all of the investigated upper soil physico‐chemical features (Table 1). In particular, in the skid trails in the recently logged cutting blocks (DIST_NEW), there was evidence of soil compaction and reduced soil organic matter content. Soil BD was about 50% higher in comparison to the values reported in the undisturbed soil and control area. Similarly, about a 40%–50% decrease in soil organic matter was observed. The magnitude of change for soil penetration resistance and soil shear resistance was even higher, with values almost doubled in DIST_NEW in comparison to the undisturbed and control treatments. Values in the skid trails in the cutting block that experienced logging 5 years before the surveys (DIST_OLD) showed the start of a recovery trend, with values that were generally significantly better than in the DIST_NEW treatment. However, for all the investigated soil physico‐chemical features, the values were still worse than in the undisturbed and control treatments, highlighting that the recovery process of the soil parameters, although started, was not completed in 5 years. Specifically, BD, penetration resistance, shear resistance and soil organic matter were +21%, +33%, +30% and −24% in comparison to the control, respectively. In contrast, we did not detect significant differences for any of the investigated soil features when comparing the soil not affected by the passage of the machine (UND_NEW and UND_OLD treatments) to the control area.

**TABLE 1:** Results of linear mixed‐effects models analysis and Tukey test (lowercase letters in ‘Group’ column) for the investigated soil physico‐chemical features.

Treatment	Average	Lower 95% CI	Upper 95% CI	Group
Soil bulk density (g cm^−3^)				
CON	0.744	0.666	0.823	c
DIST_NEW	1.104	1.026	1.182	a
DIST_OLD	0.898	0.820	0.976	b
UND_NEW	0.757	0.679	0.835	b, c
UND_OLD	0.738	0.659	0.816	c
Soil penetration resistance (MPa)				
CON	0.242	0.215	0.269	c
DIST_NEW	0.496	0.468	0.523	a
DIST_OLD	0.323	0.296	0.350	b
UND_NEW	0.242	0.215	0.269	c
UND_OLD	0.217	0.190	0.244	c
Soil shear resistance (t m^−2^)				
CON	4.51	3.30	5.72	b, c
DIST_NEW	8.19	6.98	9.40	a
DIST_OLD	5.86	4.65	7.07	b
UND_NEW	4.32	3.11	5.54	c
UND_OLD	3.88	2.66	5.09	c
Soil organic matter (%)				
CON	7.60	6.00	9.00	a, b
DIST_NEW	4.30	3.00	6.00	c
DIST_OLD	5.80	4.00	7.00	b, c
UND_NEW	8.00	7.00	9.00	a, b
UND_OLD	9.00	7.00	10.00	a

*Note:* Different lowercase letters indicate homogeneous groups at *p* < 0.05.

Abbreviations: CON, control area; DIST_NEW, soil in the skid trails in the cutting blocks harvested in 2021; DIST_OLD, soil in the skid trails in the cutting blocks harvested in 2017; UND_NEW, soil not affected by the passage of the machine in the cutting blocks harvested in 2021; UND_OLD, soil not affected by the passage of the machine in the cutting blocks harvested in 2021.

**FIGURE 2:**
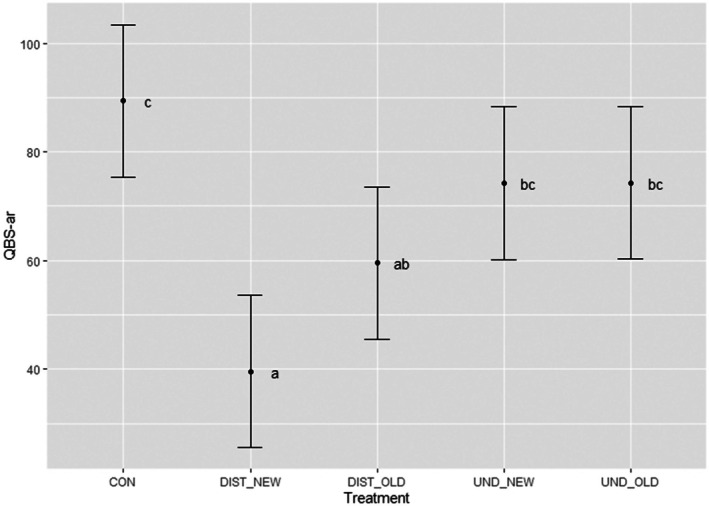
Effects of the experimental treatment on soil microarthropod biodiversity measured by the QBS‐ar index. Different lowercase letters indicate homogeneous groups at *p* < 0.05 according to the Tukey test. CON, control area; DIST_NEW, soil in the skid trails in the cutting blocks harvested in 2021; DIST_OLD, soil in the skid trails in the cutting blocks harvested in 2017; UND_NEW, soil not affected by the passage of the machine in the cutting blocks harvested in 2021; UND_OLD, soil not affected by the passage of the machine in the cutting blocks harvested in 2021.

The results concerning the effects of the experimental treatments on soil microarthropod biodiversity followed a similar trend (Figure 2). Values of the QBS‐ar index were significantly lower in the DIST_NEW treatment in comparison to both the control area and the undisturbed treatments. The magnitude of change was very high mostly in comparison to the control area, with a reduction in the values QBS‐ar of about 55%. Also, for the biodiversity of soil microarthropods the recovery process is evident in the skid trails established 5 years before the survey (DIST_OLD); values of the QBS‐ar index were higher than in DIST_NEW. However, confirming what was found for the soil physico‐chemical parameters, the values were still significantly (average magnitude of change of 33%) lower in DIST_OLD in comparison to the control area. Thus, also for this soil feature the recovery process was not complete in 5 years. Concerning the effects of the silvicultural treatment excluding the passage of the machine, measurable by comparing the undisturbed treatments with the control area, we found no significant reduction in the values of the QBS‐ar index.

### 3.2 Influence of Soil Physico‐Chemical Features on Microarthropod Biodiversity—Hypothesis d

**FIGURE 3:**
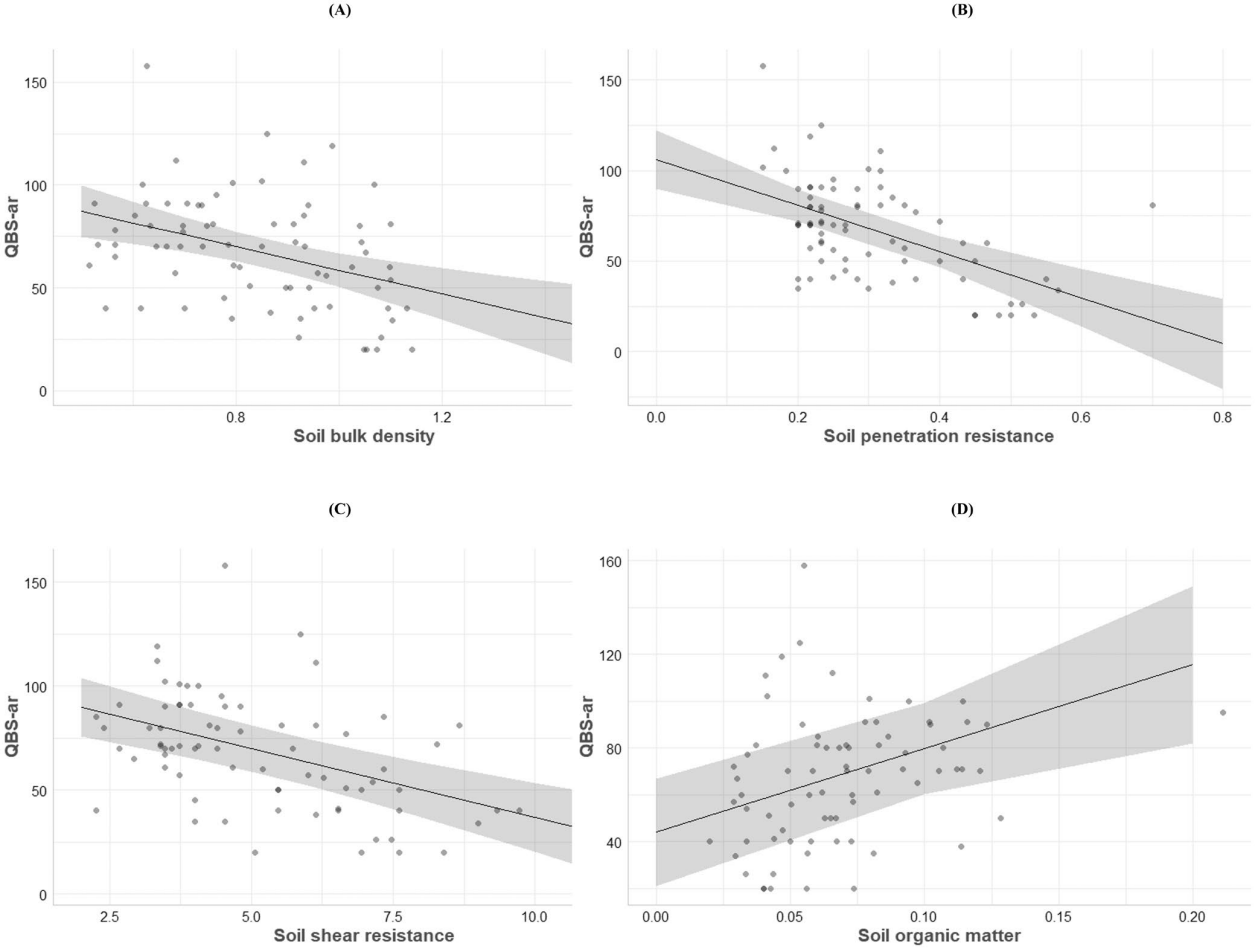
Marginal responses for (A) Linear mixed‐effects model (LMM) QBS‐ar vs. soil bulk density (g cm^−3^), (B) LMM QBS‐ar vs. soil penetration resistance (Mpa), (C) LMM QBS‐ar vs. soil shear resistance (t m^−2^), (D) LMM QBS‐ar versus soil organic matter (%). Grey dots indicate data, the black lines are regression lines, and grey ribbons represent 95% confidence intervals. For models parameters see Table 2.

Linear mixed‐effects models indicated a strong relationship between the values of the QBS‐ar index and the soil physico‐chemical features (Table 2 and Figure 3). Specifically, increased soil compaction and decreased soil organic matter content were related to low QBS‐ar values. Increasing BD from 0.8 to 1.2 g cm^−3^ caused the QBS‐ar index to decrease by about 30%. Increased soil penetration resistance from 0.2 to 0.6 MPa resulted in decreased QBS‐ar values by 47%. Increasing soil shear resistance from 2.5 to 5.0 t m^−2^ resulted in a 28% decrease in the QBS‐ar values. Increased soil organic matter from 5% to 10% resulted in increasing QBS‐ar values of about 23%. The standard deviation related to the study area (random intercept) is very limited concerning BD, soil penetration resistance and soil shear resistance; thus, the values of $R_{m}^{2}$ were very similar to $R_{c}^{2}$. In contrast, the study area‐related standard deviation for soil organic matter was higher, resulting in a substantial increase in the $R_{c}^{2}$ compared to the $R_{m}^{2}$ (Table 2).

**TABLE 2:** Linear mixed‐effects models linking QBS‐ar and soil physico‐chemical features.

QBS‐ar							
Predictors	Estimates	Standard error	*t*	*p*	Random effect standard deviation	$R_{m}^{2}$	$R_{c}^{2}$
(Intercept)	116.040	13.040	8.889	0.000	2.794	0.1675	0.1776
Soil bulk density	−57.440	14.800	−3.882	0.000			
(Intercept)	106.160	8.150	13.023	0.000	3.355	0.2717	0.2863
Soil penetration resistance	−127.410	24.018	−5.038	0.000			
(Intercept)	102.965	9.066	11.357	0.000	6.722	0.2350	0.2912
Soil shear resistance	−6.606	1.344	−4.914	0.000			
(Intercept)	43.988	11.485	3.830	0.049	12.170	0.1375	0.2967
Soil organic matter	357.761	105.416	3.394	0.001			

### 3.3 Effects of the Experimental Treatments on Litter Decomposition Rate—Hypothesis e

**FIGURE 4:**
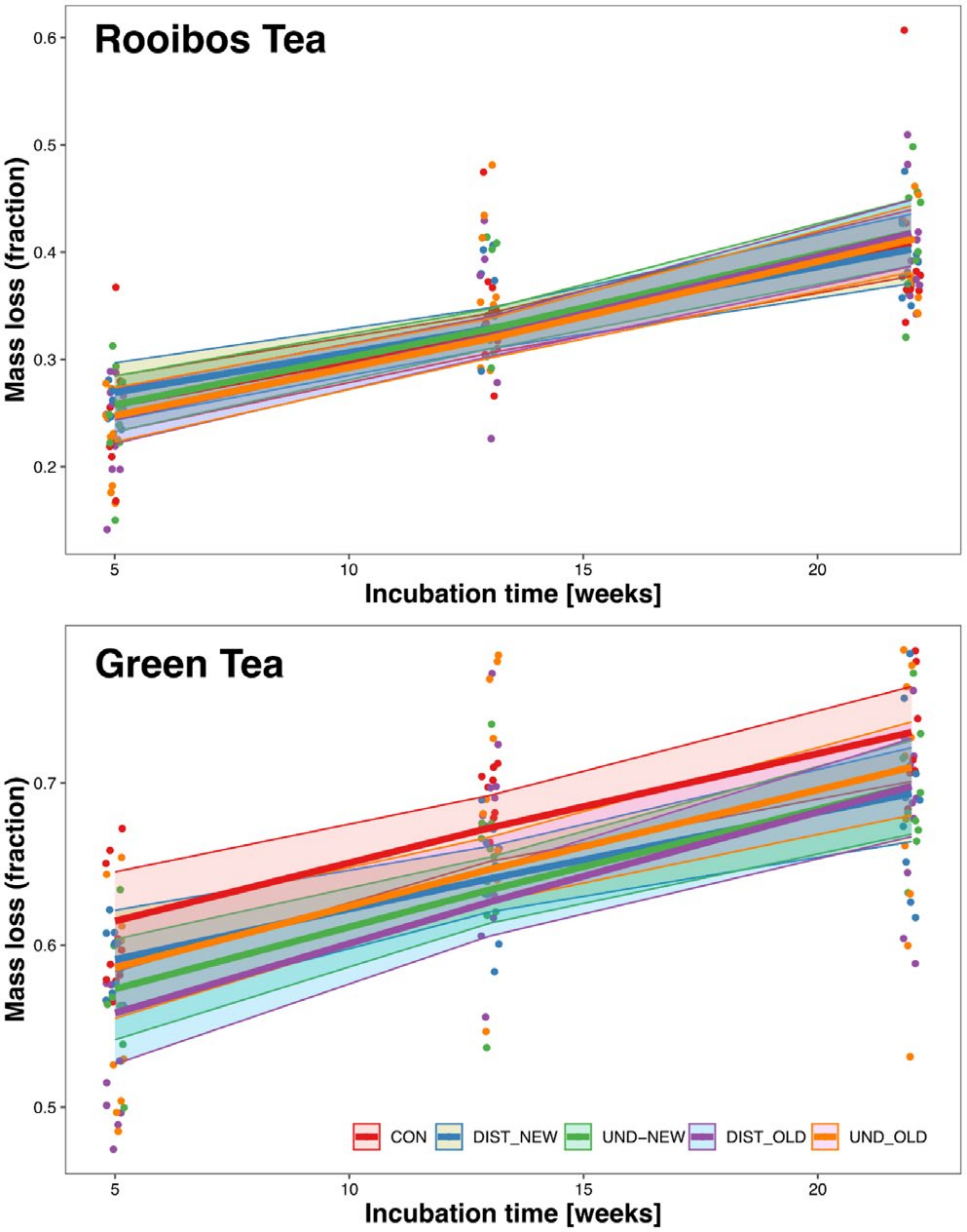
Mass loss of tea samples in a decomposition experiment on three sampling dates and among five experimental treatments, assessed using Generalised Linear Mixed‐effects models (Table 3). Dots indicate data, the lines are regression lines, and ribbons represent 95% confidence intervals. CON, control area; DIST_NEW, soil in the skid trails in the cutting blocks harvested in 2021; DIST_OLD, soil in the skid trails in the cutting blocks harvested in 2017; UND_NEW, soil not affected by the passage of the machine in the cutting blocks harvested in 2021; UND_OLD, soil not affected by the passage of the machine in the cutting blocks harvested in 2021.

Green tea mass loss depended on incubation time as indicated by a statistically significant difference among experimental treatments (Table 3 and Figure 4). Mean predicted mass loss after five, 13 and 22 weeks was 59.1% ± 6.5%, 64.1% ± 4.5% and 69.4% ± 7.0%, respectively. The maximum difference among treatments was 4.6% (62.8% ± 1.1% in DIST_OLD versus 67.7% ± 1.0% in control). Although the difference was statistically significant, only one of the treatments differed from the control at *p* < 0.05, and the magnitude of change was practically negligible. Mass loss of Rooibos tea also depended on incubation time; however, we did not find any impacts of experimental treatments (Table 3, Figure 3). Mean predicted mass loss after 5, 13 and 22 weeks was 25.8% ± 6.9%, 32.4% ± 4.3% and 40.8% ± 6.5%, respectively. The maximum difference between treatments was 0.9% (32.2% ± 0.1% in UND_OLD versus 33.1% ± 0.1% in DIST_NEW), indicating no significance of differences among experimental treatments.

**TABLE 3:** Generalised linear mixed‐effects models of mass loss in tea bags within experimental variants.

Model	Variable	Estimate	SE	*z*	Pr(> |*z*|)
Green tea	(Intercept)	0.311	0.091	3.405	0.001
AICc = −424.5	Treatment = DIST_NEW	−0.074	0.124	−0.596	0.551
AICc_0_ = −342.3	Treatment = DIST_OLD	−0.256	0.125	−2.049	0.041
Replication in site RE SD < 0.001	Treatment = UND_NEW	−0.177	0.124	−1.426	0.154
Site RE SD = 0.006	Treatment = UND_OLD	−0.124	0.125	−0.995	0.320
	Incubation time	0.031	0.006	5.007	< 0.001
	Treatment = DIST_NEW×Incubation time	−0.005	0.009	−0.581	0.561
	Treatment = DIST_OLD×Incubation time	0.004	0.009	0.479	0.632
	Treatment = UND_NEW×Incubation time	0.001	0.009	0.075	0.940
	Treatment = UND_OLD×Incubation time	0.001	0.009	0.091	0.927
Rooibos tea	(Intercept)	−1.260	0.095	−13.210	< 0.001
AICc = −434.9	Treatment = DIST_NEW	0.084	0.135	0.621	0.535
AICc_0_ = −313.7	Treatment = DIST_OLD	−0.090	0.136	−0.660	0.509
Replication in site RE SD < 0.001	Treatment = UND_NEW	−0.012	0.135	−0.090	0.928
Site RE SD = 0.043	Treatment = UND_OLD	−0.074	0.135	−0.552	0.581
	Incubation time	0.040	0.006	6.600	< 0.001
	Treatment = DIST_NEW×Incubation time	−0.005	0.009	−0.554	0.580
	Treatment = DIST_OLD×Incubation time	0.006	0.009	0.667	0.505
	Treatment = UND‐NEW×Incubation time	0.002	0.009	0.274	0.784
	Treatment = UND_OLD×Incubation time	0.004	0.009	0.471	0.638

*Note:* Control area is the reference level (intercept).

Abbreviations: AICc, Akaike's Information Criterion, corrected for small sample size; AICc_0_, AICc of null (intercept and random effects only) model; CON, control area; DIST_NEW, soil in the skid trails in the cutting blocks harvested in 2021; DIST_OLD, soil in the skid trails in the cutting blocks harvested in 2017; Pr(> |z|), *p* value; RE SD, standard deviation of random effect; SE, standard error; UND_NEW, soil not affected by the passage of the machine in the cutting blocks harvested in 2021; UND_OLD, soil not affected by the passage of the machine in the cutting blocks harvested in 2021; *z*, test statistic.

## 4 Discussion

### 4.1 Effects of the Experimental Treatments on Soil Physico‐Chemical and Biological Properties—Hypotheses a, b and c

The first hypothesis stating that *soil physico‐chemical features and soil microfauna biodiversity are significantly altered in the soil affected by the passage of the machine* was fully confirmed. In the skid trails established in late thinning in beech high forests, upper soil physico‐chemical as well as biological properties were significantly worsened in comparison to the soil not affected by the passage of the machine and to the unharvested control area (Table 1 and Figure 2). The alteration of soil features concerned not only the compaction but also the level of soil organic matter and the microarthropod biodiversity. Regarding the increase in soil compaction (BD, penetration resistance and shear resistance), the observed magnitude of change (about +50% in BD with practically doubled penetration resistance and shear resistance) is in line with previous studies investigating the effects on soil of similar machinery (cable skidder) in different silvicultural treatments (single and group selection) (Naghdi and Solgi 2014; Naghdi et al. 2016, 2023). They are also in line with a previous study assessing a different type of machinery (farm tractor with forwarding bins) in a similar silvicultural treatment but in a different geographical context (thinning in Mediterranean beech forests) (Latterini, Dyderski, et al. 2024). We therefore confirmed that the skid trails represent the areas in which logging‐induced soil disturbance is maximum. Also, the impact of logging on the forest soil is much more than mere compaction, considering that we found strongly decreased soil organic matter (about −40%) and microarthropod biodiversity (−55%) in the skid trails in the cutting block harvested in 2021.

The second research hypothesis asserting that *the soil physico‐chemical and biological features are not altered in the soil not affected by the passage of the machine* was also fully confirmed (Table 1 and Figure 2). We confirmed our previous results showing that the canopy alteration related to thinning from below in beech high forests is too low to trigger an alteration to the soil features (Latterini, Venanzi, et al. 2023; Latterini, Horodecki, et al. 2024). This can happen instead when creating a clear canopy opening such as in group selection or coppicing (Venanzi et al. 2019; Jourgholami et al. 2021).

The third hypothesis stating that a *5‐year period is not enough to restore the soil physico‐chemical and biological features of the soil in the skid trails* was fully confirmed (Table 1 and Figure 1). Although a recovery process started, highlighted by the values in the skid trails in the cutting block harvested in 2017 (DIST_OLD) which were substantially better for all the variables than in the skid trails in the 2021‐logged cutting blocks (DIST_NEW), it was not complete in 5 years. In fact, the values of all the soil features in the skid trails established 5 years before the survey (DIST_OLD), including soil microarthropod biodiversity, were significantly worse than in the undisturbed soil and control area, with magnitude of change in the order of 20%–35%. This implies that when a subsequent logging operation is carried out in the same cutting block approximately 4 years after the previous one, as is common under current management schedules, the forest soil may still be in the process of recovering from the earlier disturbance caused by machinery traffic. The soil recovery process after harvesting is a complex issue, driven by many different parameters such as the level of initial disturbance and climatic conditions (DeArmond et al. 2019, 2022, 2024). Unfortunately, this has often been neglected in the literature, with the major part of the studies focusing on short‐term disturbance (DeArmond et al. 2021). However, the recovery time is even more important than the initial disturbance. It is practically unavoidable that mechanised ground‐based logging implies a disturbance to the soil (Picchio et al. 2020). Therefore, a key consideration is determining how much time the soil needs to recover, as this information is essential for planning harvesting schedules, particularly for setting appropriate intervals between consecutive logging operations within the same cutting (Klein‐Raufhake et al. 2024).

The confirmation of the first three research hypotheses opens the topic of suggesting practical advice to address the issues that have arisen. Summarising, we found that soil disturbance to both physico‐chemical features and microarthropod biodiversity caused by thinning in temperate European beech forests is caused by the passage of the machine and not by the applied silvicultural treatment. Also, the current interval of about 4 years between two consecutive logging operations is not enough to allow for the recovery of the soil affected by the passage of the machine. The first possible solution can be to prolong the interval between two logging operations in the same cutting block. In our previous study in Mediterranean beech forests, we demonstrated that a 10‐year interval between two consecutive logging operations is enough to restore all of the upper soil properties in the skid trails (Latterini, Horodecki, et al. 2024). However, that study involved different machinery and a silvicultural approach more aligned with close‐to‐nature forestry than the one examined in this context. Therefore, dedicated trials on soil recovery time following harvesting are recommended specifically for temperate European beech forests. Alternatively, the solution should be sought in trying to decrease the magnitude of the initial disturbance, which could accelerate the recovery process (DeArmond et al. 2021). To decrease the magnitude of disturbances, there are different options to be proposed and tested in the target context. The first is shifting from ground‐based heavy machinery such as skidders to extraction systems known to cause less soil disturbance. A possible option can be aerial extraction by cable yarders, which can be particularly useful in highland beech forests, but that can also represent an option in flat terrains with low bearing capacity (Schweier and Ludowicy 2020). Nowadays, a great variety of solutions for cable yarding exists, allowing for cost‐effective forest operations in practically any scenario of topography and scale of harvesting (Leitner et al. 2023).

However, this solution can be effective in the long‐term perspective, but it is less valid in the short term, considering that loggers usually prefer extraction systems in which they are skilled. Therefore, before integrating cable yarders into silvicultural practices in temperate European beech forests, extensive training efforts are needed to raise awareness among local loggers about aerial extraction systems. The second option to decrease the magnitude of soil disturbance consists of continuing to use ground‐based extraction systems, as it is currently done, but applying some best management practices that can help reduce soil disturbance. Possible options are the use of trafficability maps to avoid the passage of the machine on areas with sensitive soil (Hoffmann et al. 2022; Latterini, Venanzi, et al. 2024; Salmivaara et al. 2024), or the application of logging mats on the soil to avoid machinery‐induced compaction (Ring et al. 2021; Labelle et al. 2022), even if this last option is not feasible when working with a skidder but it is more suitable for a forwarder. These solutions have proven to be effective in Cut‐to‐Length forest operations in the Nordic countries, but practical implementation in the reference context needs operator training and specific research to check the overall sustainability in terms of environmental, economic and social impacts (Schweier et al. 2019).

We suggest, as a future research topic, specifically testing all of the above‐mentioned alternative solutions to reduce soil disturbance in the specific context of logging operations in temperate European beech forests.

### 4.2 Influence of Soil Physico‐Chemical Features on Microarthropod Biodiversity—Hypothesis d

The fourth research hypothesis stating that *decreased soil microfauna biodiversity is related to decreased values of soil organic matter and increased soil compaction* was confirmed. We demonstrated that the decrease in soil microarthropod biodiversity that occurred in the skid trails was not only related to soil compaction, but also to the decreased level of soil organic matter (Table 2 and Figure 3). Our previous research in the context of Mediterranean forestry highlighted that soil organic matter is correlated with soil microarthropod biodiversity as much as soil compaction (Latterini, Dyderski, et al. 2024). This confirms the general knowledge about the direct relationship between good‐quality soils and the high biodiversity of soil microfauna (Menta and Remelli 2020; Sterzyńska et al. 2020). Strong reduction of food availability and loss of the soil pore system and buffering litter layer can be the major causes explaining why soil microarthropod biodiversity is significantly reduced in the skid trails, and why this decrease is driven both by compaction and organic matter removal (Wehner et al. 2021).

From an operational perspective, confirmation of the fourth hypothesis suggests that minimising disturbance to soil microfauna requires adapting extraction systems not only to reduce machinery‐induced soil compaction but also to limit overall soil displacement, which contributes to the alteration and removal of the organic soil horizon (Latterini, Spinelli, et al. 2024). From this perspective, one potential approach might involve shifting the extraction method from skidding to forwarding. Unlike skidding, where logs remain in contact with the ground during extraction, forwarding transports logs on a loading deck, minimising direct contact with the soil surface. While forwarding typically involves heavier machinery, such as forwarders, which can lead to greater soil compaction in tire‐soil contact zones (Marra et al. 2022), skidding tends to cause broader soil displacement, potentially resulting in greater disturbance to the organic soil layer (Marra et al. 2022). Given these trade‐offs, forwarding may offer a viable option for trying to reduce disturbance to the soil organic matter and, by extension, helping to conserve soil microarthropod biodiversity, particularly when combined with best management practices like the use of soil trafficability maps and logging mats to mitigate compaction (Labelle et al. 2022). However, it is important to note that this approach may be more suitable for thinning operations, as forwarders are generally not equipped to handle the large and long stems typically extracted during final shelterwood cuts. Moreover, there is currently a lack of empirical studies directly comparing the ecological impacts of forwarding versus skidding in temperate beech forests, which highlights the need for further research before drawing firm conclusions. Our previous preliminary research in the Mediterranean area did not confirm this statement (Venanzi et al. 2022). However, the study by Venanzi et al. (2022) was set up in a context where forwarders have been introduced only in recent years, thus involving loggers who were not well skilled in the application of this extraction approach. We therefore recommend specific scientific trials as future research directions.

Finally, it is worth highlighting that this study represents the first application of the QBS‐ar index in a Central European context. While the introduction of a new ecological index should always be approached with caution, our findings suggest that the QBS‐ar performed well in assessing logging‐related disturbances in temperate beech forests. The index proved to be sensitive to machinery‐induced impacts and responsive to differences in recovery time following harvesting operations.

Given these promising results, we see potential for the QBS‐ar index to be a valuable tool for evaluating soil biological quality in temperate European forestry. Its simplicity, rapid implementation, and non‐invasive nature make it particularly suitable for operational use. However, as this is a pioneering application in the region, we acknowledge that further studies are needed to validate its broader applicability, assess its limitations and compare it with other soil quality indicators under varying ecological and management conditions.

### 4.3 Effects of the Experimental Treatments on the Litter Decomposition Rate—Hypothesis e

The fifth research hypothesis stating that the *litter decomposition rate is slowed down in the skid trails and 5 years is not enough to restore the values to the pre‐harvesting levels* was rejected. The litter decomposition rate in skid trails did not show significant acceleration or slow down compared to control values and soil unaffected by machine passage (Table 3 and Figure 4).

Litter decomposition is a complex process influenced by the interplay of climatic and soil‐related (edaphic) factors (Bravo‐Oviedo et al. 2017). Silvicultural interventions and associated forest operations can modify stand structure, which in turn may affect decomposition rates (Kunhamu et al. 2009). For example, increased light penetration resulting from canopy alteration can lead to changes in the microclimate of the forest floor, particularly soil temperature and moisture, thereby influencing detritus turnover and nutrient mineralisation dynamics (Blanco et al. 2011). The magnitude of thinning has been shown to significantly affect these early‐stage decomposition rates. In general, light or moderate thinning (small reductions in canopy cover) tends to cause minor or no significant changes in litter mass loss, whereas heavy thinning (large reductions in canopy cover or basal area) can induce more pronounced changes (either accelerating or slowing decomposition depending on conditions) (Çömez et al. 2021).

Our findings can therefore be explained by the unlikeliness of the limited canopy opening resulting from thinning from below in beech forests to provide sufficient light availability to trigger altered decomposition. It can also be added that the soil disturbance caused by machine passage did not reach a level that would decrease the litter decomposition rate (Enez et al. 2015). Although the relationship between soil disturbance after ground‐based forest operations and litter decomposition rate has received some attention in the literature, our findings align with those of existing studies (Latterini, Dyderski, et al. 2023). Notably, soil compaction induced by heavy forest machinery did not lead to altered decomposition rates (Kranabetter and Chapman 1999; Yoshida et al. 2019; Latterini, Horodecki, et al. 2024). Only strong disturbances involving soil scalping significantly decreased litter decomposition rates (Enez et al. 2015). In our study areas, there was no evidence of scalping or rutting in the skid trails, even in cutting blocks harvested in 2021. Based on these findings, it appears that litter decomposition rates are primarily affected on heavily impacted skid trails, which generally represent a minor percentage of the overall skid trail network (DeArmond et al. 2021). Although our study is the first to specifically examine this topic within the target context, comprehensive and prolonged investigation, including traditional litterbag experiments, will be necessary to advance understanding in this area.

### 4.4 Study Limitations

Our selection of only two study areas with coarse‐textured soils is justified by the fact that these soils are characteristic of most Polish beech forests, ensuring the high representativeness of our findings (Poggio et al. 2021). Moreover, it is known that machinery‐induced disturbance on light texture soils is generally higher than on coarse‐texture ones (Nazari et al. 2021). Therefore, if we found such a strong disturbance on sandy and loamy soils, it is to be expected that the same logging operations in a forest located on clayey soils can trigger equal or even a higher level of disturbance. While we acknowledge that the two study areas cannot fully capture the variability of Polish beech forests, they are nonetheless highly representative of lowland and highland beech forests managed using the shelterwood method (Packham et al. 2012; Durrant et al. 2016). Thus, we believe that the obtained results are valid for the target forest typology. In any case, our results should be considered as preliminary, and further research involving more study areas is recommended to validate our findings to a larger extent.

Furthermore, our study concentrated on one specific extraction system, which is the most commonly used, though it is not the only option available. Other machinery employed in thinning beech forests includes forwarders. Future research should explore the impact of these machines on soil characteristics and litter decomposition. Additionally, it is important to note that our surveys focused on the upper soil layer (up to 5 cm depth), thereby excluding potential disturbances to the subsoil. We recommend that future research in this area should also include an assessment of deeper soil layers.

## 5 Conclusion

We investigated logging disturbance to temperate European beech forests considering physico‐chemical features, soil microarthropod biodiversity and litter decomposition rate. By applying a chronosequence approach, we also assessed the recovery time after logging, checking whether the current interval between two consecutive logging operations in the same cutting block is in line with the recovery time needed by the forest soil. Apart from the litter decomposition rate, which was not altered in any of the experimental treatments, we found a significant disturbance in terms of soil compaction, removal of organic matter and decreased microarthropod biodiversity in the soil affected by the passage of the machine. In contrast, alteration to the soil features in the soil not affected by the passage of the machine was negligible.

Our findings indicate that the recovery process for all studied variables had not yet concluded after 5 years. Skid trail sites established 5 years earlier exhibited values that continued to diverge from those of undisturbed and control areas.

We found that the decreased soil microarthropod biodiversity in the skid trails is related to both soil compaction and organic matter removal. We suggest either increasing the time interval between two consecutive logging operations in the same cutting block, or implementing a series of best management practices to decrease the magnitude of initial disturbance in the skid trails, thus accelerating the recovery process after logging.

## Author Contributions

**Francesco Latterini:** conceptualization (lead), formal analysis (lead), funding acquisition (lead), investigation (lead), methodology (lead), writing – original draft (lead), writing – review and editing (lead). **Paweł Horodecki:** investigation (supporting), writing – review and editing (supporting). **Marcin K. Dyderski:** formal analysis (equal), writing – review and editing (supporting). **Jacek Kamczyc:** investigation (supporting). **Radosław Witkowski:** investigation (supporting). **Rachele Venanzi:** investigation (supporting), writing – review and editing (supporting). **Andrzej M. Jagodziński:** writing – review and editing (supporting).

## Conflicts of Interest

The authors declare no conflicts of interest.

## Data Availability

The data are available at https://doi.org/10.5281/zenodo.14927448.

## Appendix

**TABLE A1:** Microarthropod specimens in the various soil samples.

	Treatment	Collembola	Diplura	Pseudoscorpiones	Isopoda	Chilopoda other forms EMI 20	Diplopoda forms < 5 mm EMI 20	Thysanoptera	Araneae forms > 5 mm EMI 1	Coleoptera larvae	Coleoptera	Hymenoptera	Diptera larvae	Diptera	Other holometabolous insects (larvae)	Other holometabolous insects (adults)	Hemiptera	Blattodea	Protura	Microcoryphia	Zygentoma	Opiliones	Palpigradi	Pauropoda	Symphyla	Psocoptera	Embioptera	Acari	Dermaptera (skorki)	Orthoptera (szarańczaki)
Area 1	CON					✓	✓																					✓		
	CON	✓	✓			✓			✓	✓	✓		✓						✓									✓		
	CON	✓	✓	✓	✓	✓	✓	✓	✓	✓	✓		✓				✓								✓			✓		
	CON	✓				✓				✓									✓									✓		
	CON	✓				✓		✓		✓				✓											✓			✓		
	CON	✓		✓	✓	✓		✓		✓	✓		✓				✓											✓		
	DIST_NEW	✓	✓			✓		✓	✓	✓	✓	✓																✓		
	DIST_NEW	✓																										✓		
	DIST_NEW					✓				✓																		✓		
	DIST_NEW	✓	✓					✓		✓			✓															✓		
	DIST_NEW	✓								✓																		✓		
	DIST_NEW	✓																							✓			✓		
	DIST_OLD	✓		✓				✓		✓	✓	✓																✓		
	DIST_OLD	✓		✓						✓		✓																✓		
	DIST_OLD	✓						✓		✓																		✓		
	DIST_OLD	✓								✓															✓			✓		
	DIST_OLD	✓				✓		✓																				✓		
	DIST_OLD	✓	✓					✓		✓	✓																	✓		
	UND_NEW	✓		✓				✓			✓		✓															✓		
	UND_NEW	✓	✓	✓		✓				✓				✓														✓		
	UND_NEW	✓	✓	✓		✓				✓	✓		✓												✓			✓		
	UND_NEW	✓		✓		✓			✓	✓																		✓		
	UND_NEW	✓			✓						✓																	✓		
	UND_NEW	✓		✓										✓														✓		
	UND_OLD	✓	✓	✓				✓	✓	✓																		✓		
	UND_OLD	✓	✓	✓		✓		✓		✓																		✓		
	UND_OLD	✓		✓					✓	✓																		✓		
	UND_OLD	✓	✓	✓				✓	✓	✓			✓															✓		
	UND_OLD	✓	✓	✓				✓			✓																	✓		
	UND_OLD	✓	✓	✓				✓		✓																		✓		
Area 2	CON	✓													✓				✓						✓			✓		
	CON	✓		✓		✓													✓									✓		
	CON	✓				✓				✓	✓		✓						✓									✓		
	CON	✓				✓				✓	✓		✓												✓			✓		
	CON	✓								✓		✓							✓									✓		
	CON	✓								✓			✓				✓								✓			✓		
	CON	✓				✓				✓			✓															✓		
	CON	✓							✓	✓	✓														✓			✓		
	CON	✓		✓						✓	✓		✓						✓									✓		
	DIST_NEW																											✓		
	DIST_NEW																											✓		
	DIST_NEW																											✓		
	DIST_NEW	✓											✓															✓		
	DIST_NEW																											✓		
	DIST_NEW	✓																										✓		
	DIST_NEW	✓																										✓		
	DIST_NEW																											✓		
	DIST_NEW	✓																	✓									✓		
	DIST_OLD	✓								✓		✓	✓						✓									✓		
	DIST_OLD	✓																										✓		
	DIST_OLD	✓								✓																		✓		
	DIST_OLD	✓				✓					✓		✓												✓			✓		
	DIST_OLD	✓				✓				✓									✓						✓			✓		
	DIST_OLD	✓																										✓		
	DIST_OLD	✓											✓															✓		
	DIST_OLD	✓											✓															✓		
	DIST_OLD	✓									✓														✓			✓		
	UND_NEW	✓				✓	✓			✓							✓											✓		
	UND_NEW	✓		✓		✓				✓															✓			✓		
	UND_NEW	✓				✓					✓														✓			✓		
	UND_NEW	✓								✓									✓									✓		
	UND_NEW	✓				✓				✓	✓														✓			✓		
	UND_NEW	✓				✓			✓																			✓		
	UND_NEW	✓																										✓		
	UND_NEW	✓				✓				✓																		✓		
	UND_NEW	✓								✓			✓												✓			✓		
	UND_OLD	✓				✓						✓	✓						✓									✓		
	UND_OLD	✓							✓	✓									✓									✓		
	UND_OLD	✓				✓							✓												✓			✓		
	UND_OLD	✓				✓	✓								✓													✓		
	UND_OLD	✓				✓				✓			✓												✓			✓		
	UND_OLD	✓																							✓			✓		
	UND_OLD	✓				✓				✓			✓															✓		
	UND_OLD	✓				✓																						✓		
